# Reversing the effects of ceramide imbalance via anti-ceramide immunotherapy is an effective therapy for diabetic retinopathy

**DOI:** 10.1038/s41420-025-02896-w

**Published:** 2025-12-01

**Authors:** Linbin Zhou, Jiahui Li, Changzhen Fu, Wai Kit Chu

**Affiliations:** 1 Department of Ophthalmology & Visual Sciences, The Chinese University of Hong Kong, Hong Kong SAR, China; 2 Joint Shantou International Eye Center of Shantou University and The Chinese University of Hong Kong, Shantou, China

**Keywords:** Retinal diseases, Immunotherapy

In a study recently published in *Cell Metabolism*, Dorweiler et al. defined diabetic retinopathy (DR) as a reversible ceramidopathy characteristic of an imbalance in ceramides and demonstrated the efficacy of an immunotherapeutic strategy targeting ceramides in experimental DR animal models [1]. This work sheds new light on DR pathogenesis and has important implications for developing novel and effective treatments for retarding DR progression.

DR is the most common microvascular complication of diabetes mellitus (DM) and the leading cause of vision loss in working-age populations worldwide. It is characterized by retinal ischemia, retinal inflammation, angiogenesis, and retinal vascular leakage. In the early stage of DR, which is termed non-proliferative DR (NDPR), vascular endothelial dysfunction, vascular aneurysm, and hemorrhage are prominent, while the advanced stage of DR, which is termed proliferative DR (PDR), is characteristic of neovascularization within the retina and/or macula edema. Multiple mechanisms, including inflammatory responses, oxidative stress, vascular dysfunction, and neurodegenerative alterations, are involved in DR pathogenesis. Anti-vascular endothelial growth factor (VEGF) agents targeting the pathogenic mechanisms are commonly used for DR treatments. Though it has been shown to be effective in 60% of DR patients, the rest of the DR patients either respond modestly or poorly to anti-VEGF therapies, which urgently calls for developing novel and effective treatments. On the other hand, anti-inflammation therapies are promising in DR, but the responsiveness and potential side effects are not clear. Previously, large-scale clinical trials have provided strong evidence that DR progression was strongly associated with dyslipidemia, and an intensive control of dyslipidemia markedly hindered the progression of retinal vascular dysfunction in DR [2]. Nevertheless, it remains unclear how dyslipidemia contributes to the exacerbation of retinal vascular dysfunction in DR.

Ceramide, consisting of sphingosine and an N-acylated fatty acid, is a signal of excess fatty acid in cells and a critical secondary messenger recently found to be involved in the progression of diverse metabolic diseases. Ceramide can be broadly classified into two categories: long-chain ceramides containing C14-C18-fatty acids and very-long-chain ones with C20-C26-fatty acids. A balance of ceramides between the long-chain and the very-long-chain ceramides is essential for normal cellular functions. Excess levels of long-chain ceramides can drive the progression of multiple diseases, including DM and cardiovascular diseases, and suppressing the long-chain ceramides by pharmacological inhibition or genetic manipulation disrupts disease exacerbation [3]. By contrast, decreasing very-long-chain ceramides is detrimental to cellular functions [4]. In DR, retinal vascular endothelial cell (RVEC) dysfunction is a characteristic early event exacerbating retinal tissue damages. Upregulated long-chain ceramides lead to inflammation, RVEC apoptosis, and vascular leakage in DR retinas. In contrast, generating very-long-chain ceramides stabilizes RVEC tight junctions and prevents retinal vascular leakages [5], suggesting the balance between long-chain and very-long-chain ceramides affects DR progression. However, it is still unclear about how the imbalance in ceramides leads to the dysfunction and apoptosis of RVEC and DR progression. An in-depth study on the effects of ceramide imbalance on RVEC in DR is critically important in developing novel treatments to hinder DR progression.

**Fig. 1:**
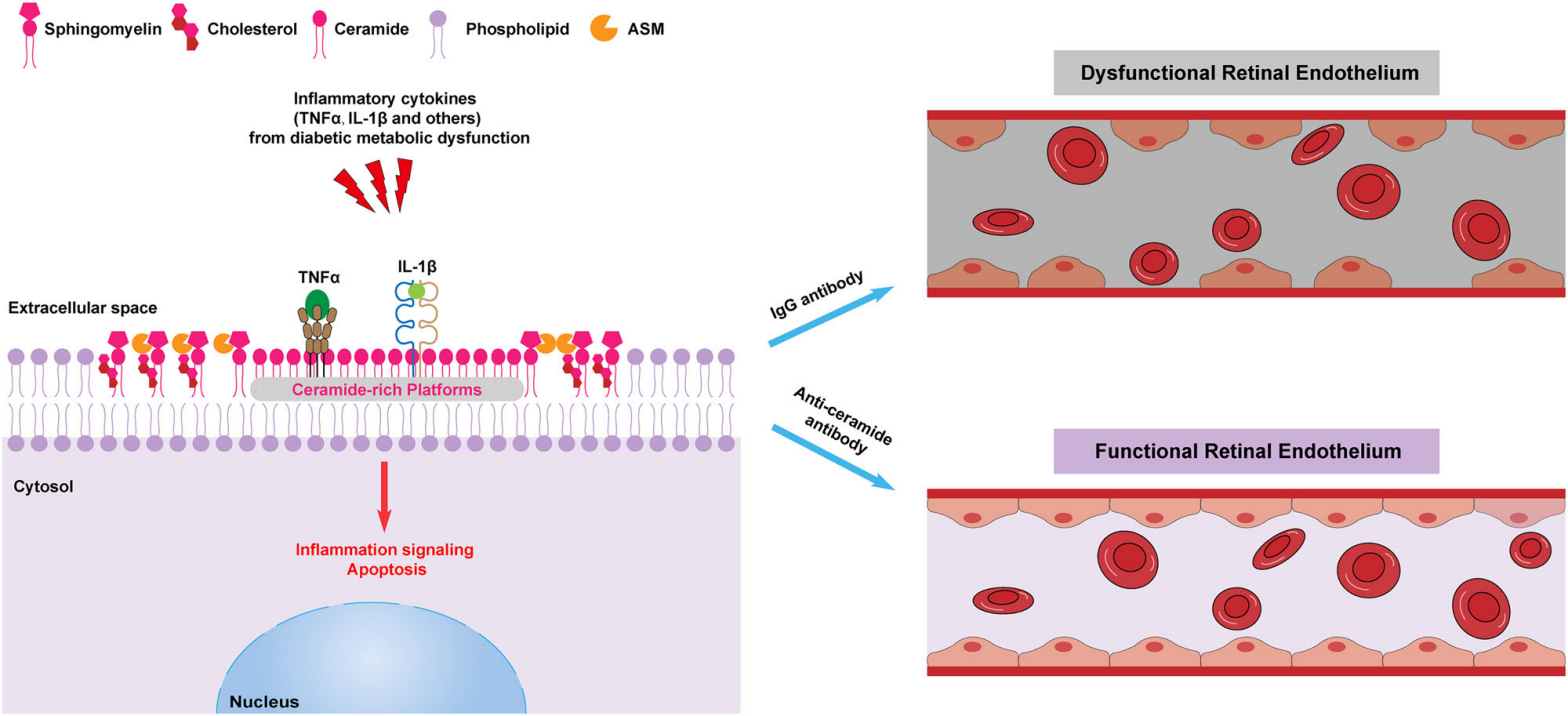
Vasculopathy in diabetic retinopathy is mediated by ceramide imbalance and can be reversed by anti-ceramide immunotherapy. Inflammatory cytokines from diabetic metabolic abnormalities, such as TNFα and IL-1β, trigger an imbalance of ceramide production, which presents as an increase in pathologic long-chain C16-ceramides synthesized by acid sphingomyelinase (ASM) and a decrease in protective very-long-chain C26-ceramides, and promotes ceramide-rich platform (CRP) formation on the plasma membrane of retinal vascular endothelial cells, thereby resulting in an escalated inflammatory signaling, endothelial cell apoptosis, retinal endothelium dysfunction, and retinal vascular leakage. Administering anti-ceramide antibodies blocks CRP formation, alleviates inflammation responses, abolishes endothelial cell apoptosis, preserves retinal endothelium function, and reverses retinal vascular leakage.

Dorweiler et al. observed an increase in vitreous C16:0/C26:0 ceramide ratio in advanced DR and demonstrated that the upregulated long-chain C16-ceramides form pro-inflammatory and pro-apoptotic ceramide-rich platforms (CRPs) on RVEC upon inflammatory cytokine stimulation and lead to retinal vascular endothelium dysfunction, which can be reversed by anti-ceramide antibodies (Fig. 1) [1]. Chronic inflammation is detrimental to RVEC functions and can lead to progressive RVEC apoptosis and vasculopathy in DR patients. To dissect the roles of ceramide imbalance in inflammation-induced RVEC dysfunction and apoptosis, the authors stimulated bovine RVEC (BRVEC) with TNFα and IL-1β, two major pro-inflammatory cytokines mediating chronic retinal inflammation in DR patients, and developed a confocal three-dimensional (3D) assay to geo-localize CRPs in real time on BRVEC. TNFα and IL-1β triggered CRPs to form within seconds by increasing long-chain C16:0 ceramides on the plasma membrane and induced apoptosis in BRVEC. Employing anti-ceramide antibodies, either 6B5 single-chain variable fragment (scFv) or m2A2, inhibited TNFα- or IL-1β-induced CRP formation and abolished apoptosis in BRVEC. These findings suggested that chronic inflammation in DR disrupted ceramide balance and promoted CRP formation in RVEC, which impaired RVEC function and survival.

Meanwhile, Dorweiler et al. further applied anti-ceramide antibodies targeting pan-ceramides to reverse ceramide imbalance-mediated vasculopathy in experimental DR animal models [1], which is a pivotal aspect of the study. The anti-ceramide antibodies could directly bind to and inactivate ceramides, which avoids affecting the de novo synthesis pathway of ceramides and thus spares potential severe detrimental side effects. Intravitreal administration of either 6B5 scFv or m2A2 anti-ceramide antibodies suppressed the expression of pro-inflammatory cytokines and cell adhesion molecules in the retina and preserved retinal vascular permeability in experimental DR models, suggesting anti-ceramide treatment is effective in hindering the progression of early NPDR into advanced PDR. Most importantly, applying these anti-ceramide antibodies to the DR animal models through subcutaneous injection, a systemic non-invasive approach, exerted similar suppressing effects on pro-inflammatory cytokine expression and vascular leakage with minimal side effects. This work highlights a safe systemic non-invasive approach for delivering anti-ceramide antibodies to effectively preventing the progression of early NPDR.

Despite these exciting findings, several critical issues remain to be addressed. First, although the authors have shown an imbalance in the vitreous ceramides of seven PDR patients in comparison with those of seven controls with macular holes, the limited sample size in this study may cause bias in interpreting the results, which requires further validation by studies with a large sample size. Second, apart from an increase in C16:0 ceramide, the C24:0 or C24:1 species were also observed to be more substantially increased in the vitreous of PDR patients than in controls. Regarding the weak evidence linking C16:0 ceramide to CRP formation, future studies focusing on the roles of other ceramide species, such as C24:0 or C24:1 species, in CRP formation in DR could be more plausible. Third, DR is a complex disease with multiple identified molecular mechanisms. Apart from retinal vascular dysfunction, it remains unknown whether ceramides are also involved in other pathological processes of DR, such as oxidative stress and retinal neurodegeneration. Last but not least, since DR is a multifactorial pathology, synergistic effects of combined treatments, such as anti-ceramide immunotherapy combined with anti-VEGF and glucose-lowering treatments, on DR deserve intense attention.

In summary, this study demonstrated that an imbalance of ceramides resulting from inflammation insults leads to abnormal CRP formation on RVEC plasma membrane, RVEC apoptosis, and vascular leakage in DR, which can be reversed by anti-ceramide immunotherapy. Future work is warranted to further validate the clinically relevant findings with a sufficient sample size and to investigate the roles of other ceramide species other than C16:0 ceramides, such as C24:0 or C24:1 ceramide species, in CRP formation in DR. It is also necessary to further study the roles of ceramide imbalance in other pathological processes of DR and potential synergistic effects of anti-ceramide immunotherapy combined with anti-VEGF and glucose-lowering treatments in DR.
